# Loneliness and Healthcare Use in Older Adults: Evidence From a Nationally Representative Cohort in Northern Ireland—A Cross-Sectional Replication Study

**DOI:** 10.3389/fpubh.2021.620264

**Published:** 2021-05-05

**Authors:** Annette Burns, Gerard Leavey, Roger O'Sullivan

**Affiliations:** ^1^Ageing Research and Development Division, The Institute of Public Health in Ireland, Dublin, Ireland; ^2^Bamford Centre for Mental Health and Wellbeing, Ulster University, Coleraine, Ireland

**Keywords:** loneliness, healthcare use, emergency department, general practice, older adults, Primary care (MeSH)

## Abstract

**Background:** Few have explored associations between loneliness and healthcare use independent of health and health behaviors. Recent indication of gender effects also requires validation across health service and cultural settings. We investigated the associations among loneliness, health and healthcare use (HCU) in older adults including stratification to investigate whether associations differed by gender.

**Methods:** Secondary analysis of a nationally representative sample of 8,309 community-dwelling adults aged 50 and over from the Northern Ireland Cohort for the Longitudinal Study of Aging. Primary outcomes were: self-reported General Practice (GP) and emergency department (ED) visits in past year. Negative binomial and logistic regression analysis were used to investigate associations between loneliness and HCU, later adjusting for potential confounders (health and health behaviors).

**Results:** Loneliness was consistently positively associated with both GP and ED visits (with IRRs ranging from 1.10 to 1.49 for GP visits, 1.16 to 1.98 for ED visits and ORs ranging from 1.13 to 1.51 for reporting at least one ED visit). With addition of health and health behaviors, all associations between loneliness and HCU became non-significant, excepting a small independent association between UCLA score and GP visits [IRR 1.03 (95% CI 1.01–1.05)]. Stratification of models revealed no gender effects.

**Conclusion:** All but one association between loneliness and HCU became non-significant when health and health behaviors were included. The remaining association was small but implications remain for health service resources at population level. No gender effects were present in contrast to recent findings in the Republic of Ireland. Further studies on gender, loneliness and healthcare use needed.

## Introduction

A number of papers have explored associations between loneliness and healthcare use (HCU) (1–11), with a subset of these controlling in some way for the potentially confounding role of health in these associations (2, 3, 5, 7, 10, 11) as well as health behaviors more recently (12). The evidence base however remains mixed with some support for the presence of significant positive associations between loneliness and HCU independent of health (mostly in relation to physician/ General Practise (GP) visits (2, 3, 5, 7, 10) but lack of associations with HCU also reported at times (3, 5, 7, 8) as well as a negative association in one study where older adults who were lonely in Singapore had significantly lower odds of physician visits (13). Our recent analysis of three waves of population level data in the Republic of Ireland, was the first to adjust for a more comprehensive picture of subjective and objective physical and mental health and to include health behaviors and, revealed an independent impact of loneliness on GP visits in older adults, which was specific to women when models were stratified by gender (12). This somewhat novel finding in relation to potential gender effects in association between loneliness and HCU as well as the still mixed evidence base on loneliness and HCU in general meant that replication of this analysis was required, especially in an alternative health service setting where healthcare access differs to the Republic of Ireland In Northern Ireland healthcare access to GPs and ED are free to all at the point of access while in the Republic of Ireland there is a mixed system of delivery with different criteria for free access, for example those over 70 are eligible for GP visit cards allowing GP visits free of charge. If one attends an emergency department without being referred by a GP however a standard fee may apply. This cross-sectional replication study will, following our previous analysis (12), be the second ever paper on loneliness and HCU, to our knowledge, to adjust for a comprehensive picture of subjective and objective physical and mental health and too also include health behaviors. This comprehensive analysis is, we feel, crucial in building evidence on the presence of true independent associations between loneliness and HCU in older adults.

We hypothesized that loneliness would again be associated with increased HCU in older adults, but that this would again be largely confounded by health (objective and subjective measures of physical and mental health including diagnoses, symptoms, and body measurements) and health behaviors (self-reported physical activity, smoking status, and alcohol consumption), with independent associations more likely for GP visits. The basis for this hypothesis was the established associations between loneliness and poorer mental and physical health (14) findings of our previous analysis (12) as well as the mixed evidence from previous studies with less comprehensive analyses for potential confounding included as above. Thus, while we expected most associations to be explained by health and health behaviors, we saw independent associations between loneliness and HCU where present as likely to be explained by either residual confounding; HCU as a form of social engagement/participation to address loneliness; or loneliness denoting seeking help for a mental/emotional health issue beyond those captured in NICOLA (depressive symptoms; and already diagnosed “psychiatric, nervous, or emotional problem”) such as anxiety symptoms. Unfortunately, it will not of course be possible within this analysis to truly infer the basis or potential drivers for any independent associations that might persist. We also hypothesized that stratifying by gender might reveal stronger independent associations in women in line with recent findings in the Republic of Ireland (12) and the known effects of gender on HCU and help seeking (15–17). The considerable literature on help seeking often focuses on factors relating to stigma and avoidance (18–20), with masculine attitudes and beliefs also thought to play a determining role in avoiding or rejecting help (21). The current paper tested these hypotheses by assessing whether loneliness was associated with HCU in a nationally representative sample of community living adults in Northern Ireland aged 50 and over; testing whether these associations, if present, were confounded by health and health behaviors; and finally investigating whether these associations and their potential confounders differed when we stratified by gender.

## Methods

### Secondary Analysis of NICOLA Data

The sample comprised participants from the Northern Ireland Cohort for the Longitudinal Study of Aging (https://nicola.qub.ac.uk/) which provides a nationally representative sample of community dwelling adults aged 50 and over living in Northern Ireland (*N* = 8,309). The current analysis is based on those aged 50 and over at Wave 1 with data collected between February 2014 and March 2016. NICOLA participants undertook a comprehensive computer-assisted home interview (CAPI) and were also invited to complete and return a self-completion questionnaire (SCQ) as well as a health assessment. An overall CAPI response rate of 63% was achieved with 59% also returning an SCQ and 44% completing a health assessment. Further detail on the sampling strategy employed in the NICOLA study can be found in a previously published report (22). Ethical approval for the study was obtained from the School of Medicine, Dentistry and Biomedical Sciences Ethics Committee, Queen's University Belfast. Access to NICOLA data is available on application through https://www.qub.ac.uk/sites/NICOLA/InformationforResearchers.

### Exposure Variables

#### UCLA Score

Loneliness was assessed in the self-completion questionnaire using the 5-item UCLA scale which is a revised version of the 20-item University of California-Los Angeles Loneliness scale ((23)). Each item is measured on a 3-point Likert scale (0–2) reflecting frequency of occurrence: “Hardly ever or never;” “Some of the time;” “Often.” Possible scores range from 0 to 10 with higher scores indicating greater loneliness.

#### UCLA Threshold

To allow comparability, a loneliness threshold variable was created in line with previous papers establishing the impact of loneliness on HCU (7, 13) whereby any participant responding “often” or “some of the time” to any of the first three items was defined as lonely.

#### Direct Item

Finally, responses of “some of the time” or “often” to UCLA item 5 which asks directly about how often respondents “feel lonely” were also modeled for associations with HCU in line with the previous cross-sectional analysis of TILDA (12) being replicated, as well as other previous analyses of loneliness and HCU which were solely based on single item measures which sought to directly assess loneliness (3, 5, 8, 10).

### Outcomes

Primary outcomes were: GP and ED visits as self-reported during computer-assisted face-to-face personal interview. A binary categorical outcome variable was also generated to capture reports of at least one ED visit in the previous 12 months given the low numbers overall and in line with the cross-sectional component of the study we were replicating (analysing the Irish Longitudinal Study of Aging) (12).

### Potential Confounders

All variables providing a profile of heath and health behaviors collected in the NICOLA study and showing significant associations with loneliness (as per Table 1) were included in our regression analyses in order to adjust for health and health behaviors as comprehensively as possible. This was in line with the cross-sectional component of the study we were replicating (analysing the Irish Longitudinal Study of Aging) (12). These were: presence of a self-reported doctor-diagnosed chronic condition (81%); reporting “troubled often with pain” (47%) or a fall in the last year (22%); BMI (mean 29.0, SD 5.22); waist circumference (mean 95.8 cm, SD 14.1); and depressive symptoms [CES-D (20-item)] (14% severe). Health behaviors included were smoking status [current (17%)/former (35%)]; self-rated alcohol consumption [drink a lot/heavily (4%); drink a moderate amount (25%)]; and physical activity with the sample split into four groups based on the number of days they reported they were moderately active in the last week (11% active 5 or more days; 8% active 3–4 days; 14% active 1–2 days; 67% active no days).

**Table 1:** Demographics, health and HCU characteristics of NICOLA cohort by Loneliness (Threshold variable based on items 1-3) (*N* = 4,717).

	**Lonely**		**Not lonely**		**t/Chi**	***P*-value**
	***n*** **=** **1,916**		***n*** **=** **2,801**			
	**41%**		**59%**			
**Gender (*****n*** **=** **4,717)**	***n***	**%**	***n***	**%**		
Male	822	38%	1,349	62%	12.7	<0.001**
Female	1,094	43%	1,452	57%		
**Age group (*****n*** **=** **4,717)**						
50–64	1,063	43%	1,411	57%	16.2	<0.001**
65–74	545	36%	948	64%		
75+	308	41%	442	59%		
**Education (*****n*** **=** **4,712)**						
Primary/none	371	40%	549	60%	0.43	0.808
Second level	845	41%	1,211	59%		
Diploma/certificate or higher	696	40%	1,040	60%		
**Marital status (*****n*** **=** **4,717)**						
Married/co-habiting	1,064	32%	2,254	68%	342.7	<0.001**
Single (never married)	192	57%	147	43%		
Separated/divorced	299	63%	176	37%		
Widowed	361	62%	224	38%		
**Healthcare use**						
GP visits past 12 months [M (SD)] (*n* = 4,673)	(3.91)	(4.72)	(2.89)	(3.83)	−8.14	<0.001**
ED visits past 12 months [M (SD)] (*n* = 4,703)	(0.37)	(1.17)	(0.23)	(0.82)	−4.75	<0.001**
ED visit (at least one) (*n* = 895/4,717)	423	47%	472	53%	20.2	<0.001**
**Doctor diagnosed chronic condition** (*n* = 3,787/4,710)	1,609	42%	2,178	58%	28.1	<0.001**
**Pain (“often troubled with”)** (*n* = 2,139/4,711)	1,047	49%	1,092	51%	112.4	<0.001**
**Fall(s) (in past year)** (*n* = 951/4,714)	500	53%	451	47%	70.3	<0.001**
**Waist cm [*****M*** **(SD)] (*****n*** **=** **2,973)**	(96.3)	(14.2)	(94.8)	(13.6)	−2.73	0.006*
**BMI [*****M*** **(SD)] (*****n*** **=** **2,983)**	(29.2)	(5.36)	(28.6)	(4.69)	−2.89	0.004*
**CES-D depressive symptoms (*****n*** **=** **2,520)**						
Severe (16+)	240	75%	79	25%	346.2	<0.001**
Moderate (8–15)	298	54%	252	46%		
None/mild (7 or less)	431	26%	1,220	74%		
**Alcohol consumption (*****n*** **=** **4,712)**						
Hardly drink/do not drink	984	43%	1,300	57%	11.7	0.008*
Drink a little	350	38%	568	62%		
Drink a moderate amount	507	38%	825	62%		
Drink a lot/heavily	73	41%	105	60%		
**Smoking (*****n*** **=** **4,714)**						
Current	313	54%	264	46%	52.5	<0.001**
Former	689	40%	1,035	60%		
Never	913	38%	1,500	62%		
**Days of moderate exercise in past week (*****n*** **=** **4,700)**						
0 days	1,259	43%	1,698	57%	15.6	0.001*
1–2 days	271	36%	482	64%		
3–4 days	155	36%	278	64%		
5–7 days	223	40%	334	60%		

* < .05; ** < .001.

Doctor diagnosed chronic conditions included in NICOLA were: cancer; chronic lung disease; cardiovascular disease (angina; high cholesterol; hypertension; diabetes; myocardial infarction or coronary thrombosis; congestive heart failure; a stroke; or TIA); asthma; arthritis; osteoporosis; Parkinson's disease; “any emotional, nervous, or psychiatric problems;” alcohol or substance abuse; Alzheimer's disease; Dementia; serious memory impairment; stomach ulcers; cirrhosis/serious liver damage; and varicose ulcers. For the purposes of this analysis the presence of at least one chronic condition was defined as answering “Yes” when asked if they were “ever told by a doctor that” they had any of the above. Those who refused to answer or reported they did not know were treated as missing.

### Statistical Analyses

Key variables and demographic characteristics of the sample were compared according to threshold and direct item loneliness using *t*-test and chi-square statistics as appropriate. As above, loneliness was modeled as (1) UCLA score; (2) UCLA threshold variable based on UCLA items 1–3; and (3) Direct item i.e., UCLA item 5 which asks directly about feeling lonely.

Multivariate negative binomial and logistic regression models were used to investigate associations between loneliness and GP visits (count) and between loneliness and ED visits (count; and any vs. none). In line with our previous analysis of HCU in another cohort (12), all models were adjusted for age, sex, education, and marital status and multilevel to account for non-independence at the household level. Potential confounders (health and health behaviors) were then simultaneously added to all models to investigate whether any independent associations between loneliness and HCU would remain. Negative binomial regression was employed for count data as an alternative to Poisson as it is useful for count data with overdispersion (i.e., sample variance is higher than the sample mean). Due to missing data the final analytic samples ranged from 2,523 to 2,466. Finally, all models were also stratified by gender to observe differences in associations according to gender.

## Results

Overall, the mean UCLA score for the 5-item scale was 2.09 (SD 2.16, Median 2) (*N* = 4,685). Among the 4,717 for whom UCLA items 1–3 were available, 41% (*n* = 1,916) were defined as lonely i.e., answered “sometimes” or “often” to at least one of these 3 items. In the case of UCLA item 5 meanwhile, or direct item loneliness, 33% (*N* = 1,605/4,818) reported they felt lonely “some of the time” (28%) or “often” (5%).

Mean self-reported GP visits in the last 12 months was 3.54 (SD 4.73, Median 2) (*N* = 8,060). Mean self-reported ED visits in the last 12 months was 0.32 (SD 1.03, Median 0) (*N* = 8,143). Overall 20.6% reported at least one ED visit.

Table 1 illustrates the main characteristics of the sample based on threshold loneliness. Meeting the “lonely” threshold was significantly related to gender, age group, marital status, GP, and ED visits as well as all health and health behavior variables. No association was found for education but since education level was related to HCU it was therefore retained in all models.

Loneliness as per UCLA item-5 was associated with all demographic, HCU health and health behavior variables.

Modeling associations between loneliness and HCU revealed that loneliness was significantly associated with both GP and ED visits across indicators of loneliness (Table 2) with IRRs ranging from 1.10 to 1.49 for number of GP visits, 1.16 to 1.98 for number of ED visits and ORs ranging from 1.13 to 1.51 in the case of reporting at least one ED visit. Following the addition of health and health behaviors to these models however, all associations between loneliness and HCU became non-significant, with one exception in the case of UCLA score and GP visits where a small independent association remained following adjustment [IRR 1.03 (95% CI 1.01–1.05)].

**Table 2:** Associations between loneliness and HCU including adjustment for health and health behaviors.

						**Adjusted for health and health behaviors**			
	**GP visits (count)**	***N***	**IRR**	**95% CI**	***P-*value**	***n***	**Adjusted IRR**	**95% CI**	***P-*value**
1	UCLA score	4,637	1.10	(1.08–1.12)	<0.001**	2,466	1.03	(1.01–1.05)	0.013*
Men:		2,144	1.10	(1.07–1.13)	<0.001**	1,185	1.03	(0.99–1.06)	0.143
Women:		2,493	1.10	(1.07–1.13)	<0.001**	1,281	1.03	(1.00–1.05)	0.056
2	UCLA Threshold	4,669	1.35	(1.25–1.46)	<0.001**	2,479	1.04	(0.95–1.14)	0.404
Men:		2,160	1.31	(1.17–1.47)	<0.001**	1,193	0.97	(0.85–1.11)	0.649
Women:		2,509	1.39	(1.26–1.53)	<0.001**	1,286	1.10	(0.98–1.23)	0.091
3	Direct Item	4,767	1.49	(1.37–1.61)	<0.001**	2,510	1.05	(0.96–1.16)	0.294
Men:		2,210	1.57	(1.38–1.79)	<0.001**	1,211	1.03	(0.89–1.21)	0.665
Women:		2,557	1.44	(1.30–1.59)	<0.001**	1,299	1.06	(0.94–1.19)	0.352
	**ED visits (count)**	***N***	**IRR**	**95% CI**	***P*****-value**	***n***	**Adjusted IRR**	**95% CI**	***P-*****value**
1	UCLA score	4,669	1.16	(1.11–1.22)	<0.001**	2,476	1.03	(0.96–1.09)	0.411
Men:		2,152	1.15	(1.09–1.22)	<0.001**	1,192	0.99	(0.92–1.08)	0.922
Women:		2,517	1.18	(1.11–1.25)	<0.001**	1,284	1.07	(0.98–1.17)	0.130
2	UCLA Threshold	4,700	1.56	(1.29–1.89)	<0.001**	2,489	0.95	(0.75–1.21)	0.674
Men:		2,168	1.39	(1.08–1.78)	0.010*	1,200	0.86	(0.61–1.22)	0.405
Women:		2,532	1.75	(1.36–2.25)	<0.001**	1,289	1.12	(0.84–1.49)	0.444
3	Direct Item	4,802	1.98	(1.63–2.39)	<0.001**	2,520	1.23	(0.97–1.54)	0.083
Men:		2,219	2.01	(1.56–2.58)	<0.001**	1,218	1.21	(0.85–1.73)	0.289
Women:		2,583	1.93	(1.49–2.51)	<0.001**	1,302	1.23	(0.92–1.66)	0.168
	**ED visit (1 or more)**	***N***	**ORR**	**95% CI**	***P-*****value**	***n***	**Adjusted ORR**	**95% CI**	***P-*****value**
1	UCLA score	4,680	1.13	(1.09–1.17)	<0.001**	2,479	1.02	(0.96–1.09)	0.457
Men:		2,155	1.13	(1.07–1.19)	<0.001**	1,192	1.00	(0.92–1.10)	0.924
Women:		2,525	1.14	(1.07–1.20)	<0.001**	1,287	1.04	(0.96–1.13)	0.349
2	UCLA Threshold	4,712	1.38	(1.18–1.61)	<0.001**	2,492	0.98	(0.77–1.26)	0.889
Men:		2,171	1.31	(1.04–1.66)	0.021*	1,200	0.99	(0.69–1.43)	0.971
Women:		2,541	1.44	(1.17–1.76)	0.001*	1,292	0.99	(0.70–1.38)	0.933
3	Direct Item	4,813	1.51	(1.2–1.78)	<0.001**	2,523	1.05	(0.81–1.36)	0.700
Men:		2,222	1.65	(1.28–2.12)	<0.001**	1,218	1.07	(0.70–1.63)	0.762
Women:		2,591	1.44	(1.17–1.77)	0.001*	1,305	1.08	(0.77–1.52)	0.666

All models adjusted for age, sex, education, and marital status. * < .05; ** < .001.

The stratification of models by gender revealed no gender effects in relation to associations between loneliness and HCU in this cohort.

## Discussion

Loneliness was consistently positively associated with number of GP visits and number of ED visits in the past year, with IRRs ranging from 1.10 to 1.49 and 1.16 to 1.98, respectively. There was also a positive association between all three indicators of loneliness and reporting at least one ED visit in the past year with ORs ranging from 1.13 to 1.51 in the sample overall. Upon adjusting for health and health behaviors, loneliness no longer had any impact on ED visits in terms of number of visits or reporting at least one visit compared to none. In relation to GP visits, associations became non-significant for two of the three indicators of loneliness (UCLA threshold and direct item loneliness) but in the case of UCLA score a small association independent of health and health behaviors persisted in the sample overall [IRR 1.03 (95% CI 1.01–1.05)]. Thus, those with higher loneliness scores on the UCLA seem to report visiting their GP a little more often irrespective of their health and health behaviors. The stratification of models by gender did not reveal the presence of any gender effects. In line with our first hypothesis therefore loneliness was associated with increased HCU, with these associations largely explained by health and health behaviors. In contrast to our second hypothesis however, and recent findings elsewhere (12), no gender effects were present in associations between loneliness and HCU.

The fact that the associations between loneliness and HCU found in this cross-sectional replication study were again small and largely explained by health and health behaviors, supports the importance of a comprehensive adjustment for potential confounding when investigating impacts of loneliness on HCU. Health and health behaviors, which have long been known to be associated with both loneliness and HCU are clearly key confounders of these associations and future studies on loneliness and HCU need to include these in order to provide a clearer picture of independent associations. While all associations with ED visits disappeared once health and health behaviors were accounted for there was still a small significant independent association with GP visits in the sample overall implying again that impacts of loneliness on HCU independent of health where present appear to be found in general practice rather than emergency department settings (2, 3, 7, 10, 12). In line with previous studies the size of the independent association found was small (1, 9) and while there are implications for health services resources at a population level, overall, it was clear that loneliness is not a major driver of additional HCU among older adults in Northern Ireland. Rather, in line with our hypothesis, the association between loneliness and HCU was largely explained by co-occurring poorer health. This is unsurprising given how frequently the associations between loneliness and poorer physical and mental health and health behaviors have previously been demonstrated in the literature already (14, 24–26).

The presence of associations between loneliness and GP visits in this analysis however, even if largely non-independent, demonstrates the fact that lonely older adults are likely to see their GP regularly, and thus provides further support for the primary care setting as a potential opportunity for an assessment of loneliness to become as routine as checking weight, blood pressure, alcohol consumption, smoking and diet as well as a point from which to redirect toward appropriate services and tailored resources where present. This assessment could be done using a brief validated measure such as the 3-item UCLA (27) given time constraints and the seeming inaccuracy of perception (28). In addition to the selection of a standardized assessment tool however, training and referral pathways will also be required to facilitate a useful response. Unfortunately, the evidence to date on effective interventions for loneliness remains greatly limited with further high-quality studies involving large samples and diverse populations needed (29, 30).

The lack of gender effects observed here may mean that the findings of our previous analysis of TILDA represent a spurious finding (12). Clearly further research is needed to clarify the existence of any consistent gender effects in loneliness and HCU, including in other jurisdictions. This is further complicated by evidence that men and women may report loneliness differently (31).

This cross-sectional replication also provided an opportunity to test these associations in an alternative health service setting, where healthcare is free to all at the point of access. Given the findings of the current study were comparable to our analysis of the Republic of Ireland (12) in terms of the sample overall, the differing access provided by these health service settings does not appear to explain overall associations. In short, while one might expect free healthcare would normally motivate more non-medical visits, this did not appear to be the case. The question that remains unanswered and outside the scope of this paper is why there is a perception that lonely older adults are more likely to visit GPs and A&Es regardless of health, it does seem in part to be based on stereotypical assumptions and views on who is lonely and does require further consideration (32).

### Strengths and Limitations

This analysis is strengthened by the population representative sample of community dwelling adults aged 50 and over in Northern Ireland which the NICOLA study provided. The fact that the NICOLA study was modeled so closely on the design of the Irish Longitudinal Study of Aging (TILDA) further strengthened this as a cross-sectional replication of our analysis of the TILDA data. Thus, the majority of variables included in our previous study were replicated exactly in the NICOLA study with data also collected in the same way (*via* CAPI; SCQ and Health assessment) meaning a precise cross-sectional replication allowing comparison of population level data for respondents with a differing health service was possible. Finally, to our knowledge, this study is the second to date to explore the role of gender in associations between loneliness and HCU and confounding by health. It is also only the second analysis of loneliness and HCU to include health behaviors.

Limitations to the current analysis included the cross-sectional nature as only one wave of NICOLA data has been made available to date. A CAPI weight as applied in our TILDA analysis was also not yet available in NICOLA. Some minor differences in the data available were also present with NICOLA lacking equivalent data in relation to the presence of cataracts, glaucoma, or age-related macular degeneration which were included as 3 of the 26 possible doctor diagnosed chronic conditions included in defined presence of at least one condition in our previous TILDA analysis. Also missing was a scale assessing anxiety symptoms. In the absence of the IPAQ and CAGE measures substitutions were made in assessing physical activity and alcohol problem and models were adjusted for age group rather than continuous age. Similar to previous papers on loneliness and HCU where only cross-sectional data were available, this study was also somewhat limited by the way in which data were collected. Namely and as in previous papers on this topic, the outcomes, HCU, were recalled for previous 12 months while loneliness had been reported in relation to the present moment during data collection (3, 10). This is a clear limitation and must be taken into account in interpreting the findings of this and previous cross-sectional analyses (3, 10, 12). Some reassurance is provided however by the very similar patterns of results revealed both cross-sectionally and longitudinally in the prior TILDA analysis where data was collected in the same way (12). Nonetheless, further research is clearly needed, and future analyses should continue to seek to address this limitation by employing prospective data while also fully adjusting for health and health behaviors.

### Conclusion

This population level study sought to replicate the recent cross-sectional analysis of loneliness and HCU in older adults in the Republic of Ireland adjusting for health and health behaviors as well as investigating the role of gender in associations. Consistent associations between loneliness and HCU which largely disappeared once health and health behaviors were accounted for were again observed. A single small association between UCLA score and GP visits persisted regardless of health and health behaviors, indicating that those with higher loneliness scores report visiting their GP a little more often independent of their health and health behaviors. Contrary to recent findings in the Republic of Ireland, no gender effects were present.

## Data Availability Statement

The data analyzed in this study is subject to the following licences/restrictions: The sample comprised participants from the Northern Ireland Cohort for the Longitudinal Study of Aging (https://nicola.qub.ac.uk/). Data is available upon application to the NICOLA team. Requests to access these datasets should be directed to nicola-research@qub.ac.uk.

## Ethics Statement

This was a secondary analysis of the NICOLA study which involved human participants. Ethical approval for the NICOLA study was obtained from the School of Medicine, Dentistry and Biomedical Sciences Ethics Committee, Queen's University Belfast prior to data collection. Written informed consent for participation was not required for this study in accordance with the national legislation and the institutional requirements.

## Author Contributions

AB conceived the idea for the study, analyzed the data, and wrote the first draft of the manuscript. GL provided supervision, aided the writing process, and structure of the manuscript. RO'S was the principal investigator for this project, supervised the project, provided feedback on the design, the analysis, the structure of the manuscript, and assisted in review of the current paper. All authors read and approved the final manuscript.

## Conflict of Interest

The authors declare that the research was conducted in the absence of any commercial or financial relationships that could be construed as a potential conflict of interest.
